# Circulating Concentrations of GDF11 are Positively Associated with TSH Levels in Humans

**DOI:** 10.3390/jcm8060878

**Published:** 2019-06-19

**Authors:** Juan Añón-Hidalgo, Victoria Catalán, Amaia Rodríguez, Beatriz Ramírez, Adrián Idoate-Bayón, Camilo Silva, Carmen Mugueta, Juan C. Galofré, Javier Salvador, Gema Frühbeck, Javier Gómez-Ambrosi

**Affiliations:** 1 Metabolic Research Laboratory, Clínica Universidad de Navarra, 31008 Pamplona, Spain; janon@alumni.unav.es (J.A.-H.); vcatalan@unav.es (V.C.); arodmur@unav.es (A.R.); bearamirez@unav.es (B.R.); aidoate.10@alumni.unav.es (A.I.-B.); gfruhbeck@unav.es (G.F.); 2 CIBER Fisiopatología de la Obesidad y Nutrición (CIBEROBN), Instituto de Salud Carlos III, 31008 Pamplona, Spain; csilvafr@unav.es (C.S.); jsalvador@unav.es (J.S.); 3 Obesity and Adipobiology Group, Instituto de Investigación Sanitaria de Navarra (IdiSNA), 31008 Pamplona, Spain; 4 Department of Endocrinology and Nutrition, Clínica Universidad de Navarra, 31008 Pamplona, Spain; jcgalofre@unav.es; 5 Department of Biochemistry, Clínica Universidad de Navarra, 31008 Pamplona, Spain; cmugueta@unav.es

**Keywords:** GDF11, TSH, REE, leptin, CRP

## Abstract

Growth differentiation factor 11 (GDF11) is a member of the transforming growth factor (TGF)-β superfamily which declines with age and has been proposed as an anti-aging factor with regenerative effects in skeletal muscle in mice. However, recent data in humans and mice are conflicting, casting doubts about its true functional actions. The aim of the present study was to analyze the potential involvement of GFD11 in energy homeostasis in particular in relation with thyroid hormones. Serum concentrations of GDF11 were measured by enzyme-linked immunosorbent assay (ELISA) in 287 subjects. A highly significant positive correlation was found between GDF11 and thyroid-stimulating hormone (TSH) concentrations (*r* = 0.40, *p <* 0.001). Neither resting energy expenditure (REE) nor REE per unit of fat-free mass (REE/FFM) were significantly correlated (*p* > 0.05 for both) with GDF11 levels. In a multiple linear regression analysis, the model that best predicted logGDF11 included logTSH, leptin, body mass index (BMI), age, and C-reactive protein (logCRP). This model explained 37% of the total variability of logGDF11 concentrations (*p <* 0.001), with only logTSH being a significant predictor of logGDF11. After segregating subjects by TSH levels, those within the low TSH group exhibited significantly decreased (*p <* 0.05) GDF11 concentrations as compared to the normal TSH group or the high TSH group. A significant correlation of GDF11 levels with logCRP (*r* = 0.19, *p* = 0.025) was found. GDF11 levels were not related to the presence of hypertension or cardiopathy. In conclusion, our results show that circulating concentrations of GDF11 are closely associated with TSH concentrations and reduced in subjects with low TSH levels. However, GDF11 is not related to the regulation of energy expenditure. Our data also suggest that GDF11 may be involved in the regulation of inflammation, without relation to cardiac function. Further research is needed to elucidate the role of GDF11 in metabolism and its potential involvement in thyroid pathophysiology.

## 1. Introduction

Thyroid hormones are involved in the regulation of basal metabolic rate and are active players in many physiological processes, such as growth, development, as well as energy expenditure [1]. It has been shown that basal metabolism is very sensitive to thyroid hormones, with the dysregulation of the thyroid axis leading to marked alterations in energy balance [1,2]. The thyroid gland is regulated by the hypothalamic thyrotropin-releasing hormone, which regulates the secretion of thyroid-stimulating hormone (TSH) from the pituitary gland [2]. Activation of thyroxine (T_4_), to the active form, triiodothyronine (T_3_), by 5′-deiodinase type 2 is a key cellular mechanism in the regulation of metabolism in peripheral tissues [2]. Thyroid hormone signaling takes place peripherally, mostly in the liver, white and brown adipose tissues, but also centrally, in the hypothalamus [1,2]. Thyroid hormones directly stimulate energy expenditure changing the functionality and increasing the expression of genes involved in thermogenesis, such as uncoupling protein 1, in peripheral tissues such as skeletal muscle and brown adipose tissue (BAT), but also through central actions [3].

The transforming growth factor (TGF)-β superfamily of signaling proteins encompasses more than 30 members exerting pleiotropic functions through similar pathways of signal transduction [4,5,6]. At least three subfamilies have been described to date: TGF-βs, bone morphogenetic proteins (BMPs)/growth differentiation factors (GDFs), and activins/inhibins [5,6]. GDF11 is expressed in many tissues and organs including skeletal muscle, heart, pancreas, kidney, intestine, developing nervous system, olfactory system, as well as in the retina [7]. GDF11 levels have been shown to decline with aging in mice losing their reported actions to promote skeletal muscle regeneration [8]. However, further studies have found contradictory results showing that GDF11 may produce skeletal muscle atrophy rather than regeneration [6,7]. In humans, results regarding circulating levels of GDF11 are conflicting, showing a decrease [9], an increase or a trend to an increase [10,11], or no change [11,12] with aging. We have recently shown that GDF11 levels decline with aging in humans with no influence on skeletal muscle mass [13]. Recent data suggest that another member of the TGF-β superfamily, GDF15, correlates with thyroid hormones being elevated in patients with hyperthyroidism, and that the treatment with thyroid hormone increases the expression of GDF15 in BAT in mice [14]. Therefore, we hypothesized that GDF11 could be involved in energy expenditure and associated with thyroid hormones.

We aimed to investigate in humans the relation of GDF11 with thyroid hormones and energy expenditure in a large cohort of well-characterized subjects. In addition, we studied the levels of GDF11 in individuals with high, normal, or low levels of TSH.

## 2. Materials and Methods

### 2.1. Study Participants

We conducted a cross-sectional analysis of 287 patients (121 men and 166 women) aged 18–79 years, with similar socioeconomic characteristics, including patients visiting the Department of Endocrinology and Nutrition of the Clínica Universidad de Navarra (Pamplona, Spain) for weight loss treatment as well as hospital and University staff undergoing an annual routine health check-up. In a subsequent analysis, individuals with complete thyroid profile (*n* = 128) were classified according to TSH concentrations in high TSH (TSH > 4.7 μU/mL), normal TSH (TSH between 0.5–4.7 μU/mL), or low TSH (TSH < 0.5 μU/mL) based on the normal reference values [15]. Participants underwent a clinical assessment including medical history, physical examination, body composition analysis, and comorbidity evaluation performed by a multidisciplinary consultation team. Individuals with signs of infection were excluded. The experimental design was approved by the Research Ethics Committee of the University of Navarra (protocol 2017.121) and the study was performed in accordance with the ethical standards as laid down in the Declaration of Helsinki and its later amendments. Volunteers gave their informed consent to participate in the study.

### 2.2. Anthropometry and Resting Energy Expenditure

The anthropometric and body composition determinations as well as the blood extraction were performed in a single day. Height was measured to the nearest 0.1 cm with a Holtain stadiometer (Holtain Ltd., Crymych, UK), while body weight was measured with a calibrated electronic scale to the nearest 0.1 kg with subjects wearing a swimming suit and cap. Body mass index (BMI) was calculated as weight in kg divided by the square of height in meters. Waist circumference was measured at the midpoint between the iliac crest and the rib cage on the midaxillary line. Blood pressure was measured after a 5-minute rest in the semi-sitting position with a sphygmomanometer. Blood pressure was determined at least three times at the right upper arm and the mean was used in the analyses. Body density was estimated by air displacement plethysmography (Bod-Pod^®^, Life Measurements, Concord, CA, USA). Percentage of body fat was estimated from body density using the Siri equation as previously described [16]. Fat-free mass (FFM) index (FFMI) was calculated as FFM in kg divided by the square of height in meters [17]. Resting energy expenditure (REE) was measured through indirect calorimetry (Vmax29, SensorMedics Corporation, Yorba Linda, CA, USA) [18].

### 2.3. Serum Biochemistry

Blood samples were collected after an overnight fast in the morning in order to avoid potential confounding influences due to hormonal rhythmicity. Plasma glucose was analyzed by an automated analyzer (Roche/Hitachi Modular P800, Basel, Switzerland) as previously described [19]. Insulin was measured by means of enzyme-amplified chemiluminescence assay (IMMULITE 2000, Siemens AG, Erlangen, Germany). The homeostatic model assessment (HOMA) was used as an indirect measure of insulin resistance. Total cholesterol and triglyceride concentrations were determined by enzymatic spectrophotometric methods (Roche, Basel, Switzerland). Serum high-density lipoprotein cholesterol (HDL-C) was quantified by a colorimetric method in a Beckman Synchron^®^ CX analyzer (Beckman Instruments, Ltd., Bucks, UK). Low-density lipoprotein (LDL-C) was calculated by the Friedewald formula. High-sensitivity C-reactive protein (CRP) was measured using the Tina-quant CRP (Latex) ultrasensitive assay (Roche). Uric acid and creatinine were measured by enzymatic tests (Roche) in an automated analyzer (Roche/Hitachi Modular P800, Basel, Switzerland). TSH, free thyroxine (fT_4_), and free triiodothyronine (fT_3_) concentrations were measured by an electro-chemiluminescence immunoassay (ECLIA) using Roche Elecsys^®^ E170 immunoassay analyzer (Roche, Basel, Switzerland). Leptin levels were quantified by a double-antibody radioimmunoassay (RIA) method (Linco Research, Inc., St. Charles., MO, USA) as previously described [18,20]; intra- and interassay coefficients of variation were 5.0 and 4.5%, respectively. Thyroid peroxidase (anti-TPO) and thyroglobulin (anti-TG) antibodies were measured by enzyme-linked immunosorbent assay (ELISA) (QUANTA Lite^®^, Inova Diagnostics, San Diego, CA, USA). Serum GDF11 concentrations were determined using a validated ELISA kit (Human GDF11 ELISA kit, E01G0124, BlueGene Biotech, Shanghai, China) with intra- and interassay coefficients of variation being 5.5 and 7.8%, respectively. According to the manufacturer, no cross reactivity has been observed with any other analogue.

### 2.4. Cardiac Function

Resting electrocardiogram (ECG) in individuals with thyroid profile was recorded on a PageWriter TC20 Cardiograph (Philips Medical Systems, Andover, MA, USA). In a subset of patients (*n* = 40), Doppler ultrasound measurements were obtained with a Philips SONOS 7500 Echocardiography System (Philips Medical Systems, Andover, MA, USA).

### 2.5. Statistical Analysis

Data are presented as mean ± SD unless otherwise indicated. Normal distribution was assessed by Kolmogorov–Smirnov test. Differences between groups were analyzed by one-way ANOVA followed by Fisher’s least significant difference (LSD) tests or two-tailed unpaired Student’s *t* tests, as appropriate. Correlations between two variables were computed by Pearson’s correlation coefficients (*r*). Multivariate linear regression analysis was conducted for the dependent variable logGDF11 including the variables which showed a significant correlation with logGDF11 as independent variables. The calculations were performed using SPSS 23 (SPSS, Chicago, IL, USA) and GraphPad Prism 6 (GraphPad Software, Inc., La Jolla, CA, USA). A *p* value lower than 0.05 was considered statistically significant.

## 3. Results

**Figure 1:**
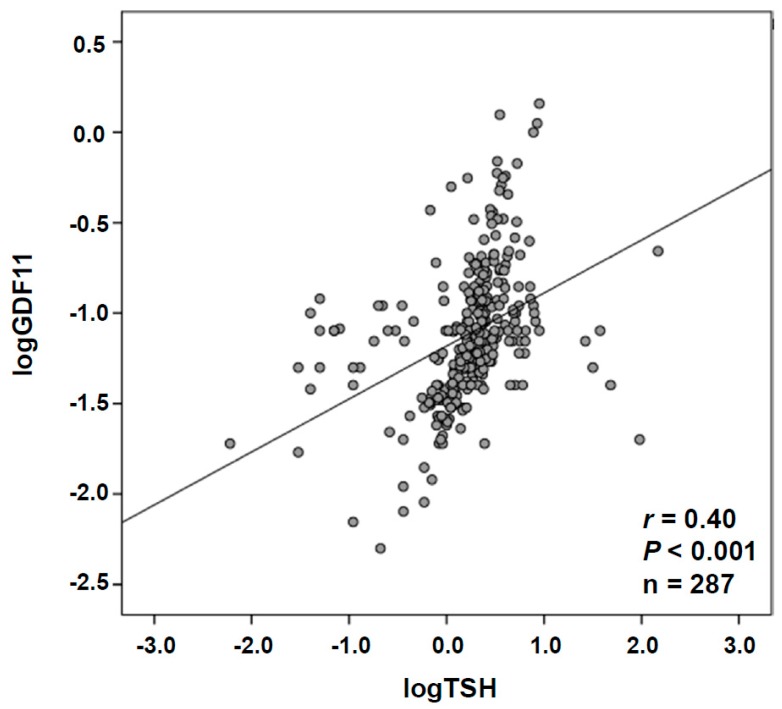
Association of growth differentiation factor 11 (GDF11) concentrations with thyroid-stimulating hormone (TSH) levels. Scatter diagram showing the correlation between the circulating concentrations of GDF11 and TSH. Pearson’s correlation coefficient and *p* value are indicated. The line of adjustment is shown.

Anthropometric and biochemical characteristics of the individuals included in the study are shown in Table 1. From the whole cohort, 121 (42%) were males and 166 (58%) were females. No significant differences regarding gender in circulating concentrations of GDF11 (males 0.129 ± 0.199, females 0.114 ± 0.148 ng/mL; *p* = 0.986) or TSH (males 2.5 ± 2.8, females 4.4 ± 14.3 ng/mL; *p* = 0.794) were observed. Interestingly, a highly significant positive correlation was found between GDF11 and TSH levels (*r* = 0.40, *p <* 0.001) (Figure 1 and Table 2). Since GDF11 is influenced by age [13], we reassessed the correlation analysis adjusting by age. The correlation between GDF11 and TSH levels was found almost unchanged (*r* = 0.38, *p <* 0.001) after adjusting by age (Table 2). Moreover, a negative correlation with age (*r* = −0.16, *p* = 0.006) and a positive one with BMI (*r* = 0.15, *p* = 0.009) and waist circumference (*r* = 0.13, *p* = 0.029) were observed. The correlations of GDF11 with BMI and waist circumference were conserved after adjustment by age. Neither REE (*r* = 0.10, *p* = 0.149) nor REE per unit of FFM (REE/FFM) (*r* = 0.02, *p* = 0.725, Table 2) were significantly correlated with GDF11, while REE/FFM was significantly correlated with leptin concentrations (*r* = 0.33, *p <* 0.001). Finally, we found significant correlations of GDF11 levels with CRP (*r* = 0.19, *p* = 0.025) and leptin (*r* = 0.14, *p* = 0.027) levels that were maintained after adjustment by age for both CRP (*r* = 0.18, *p* = 0.038) and leptin (*r* = 0.14, *p* = 0.026). In the multiple linear regression analysis (Table 3), the model that best predicted logGDF11 included logTSH, leptin, BMI, age, and logCRP. This model explained 37% of the total variability of logGDF11 concentrations (*p <* 0.001), with only logTSH being a significant predictor of logGDF11.

**Figure 2:**
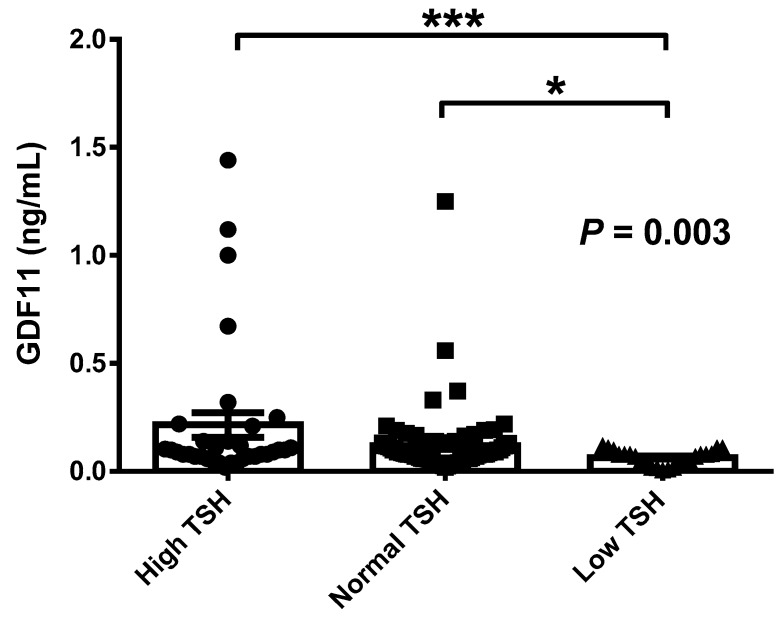
Serum GDF11 concentrations in subjects with high (*n* = 32), normal (*n* = 76), and low (*n* = 20) concentrations of TSH. Bars represent mean ± SEM. Statistical differences between groups were analyzed by one-way ANOVA followed by Fisher’s least significant difference (LSD) tests. * *p <* 0.05 and *** *p <* 0.001. GDF11 concentrations were logarithmically transformed for statistical analysis due to their non-normal distribution.

We next stratified the 128 participants with thyroid profile into high (>4.7 μU/mL), normal (0.5–4.7 μU/mL), or low (<0.5 μU/mL) TSH levels (Table 4). No significant differences in anthropometric variables, including FFM (*p* = 0.797) and FFMI (*p* = 0.534) were observed. A significant difference in serum total cholesterol (*p* = 0.018) and a tendency in LDL-cholesterol (*p* = 0.069) with reduced levels in the low-TSH group were found. No other significant differences between groups were observed except for TSH and fT_4_ (both *p <* 0.001), as expected. Subjects in the low TSH group exhibited significantly decreased GDF11 concentrations as compared to the normal TSH group (0.068 ± 0.035 vs. 0.120 ± 0.155 ng/mL; *p* = 0.019) or the high TSH group (0.224 ± 0.341 ng/mL; *p <* 0.001) as illustrated in Figure 2. In this subsample of 128 subjects, GDF11 and TSH levels showed again a highly significant positive correlation (*r* = 0.30, *p <* 0.001), while no correlation was observed between GDF11 and fT_4_ (*r* = −0.06, *p* = 0.517) or fT_3_ levels (*r* = −0.10, *p* = 0.366). REE was significantly correlated with fT_4_ levels (*r* = 0.23, *p* = 0.022). From the 128 patients, 23 were on L-thyroxine treatment. No effect of L-thyroxine medication on GDF11 levels was observed (with 0.140 ± 0.287, without 0.120 ± 0.141 ng/mL; *p* = 0.644). No significant differences in GDF11 concentrations were observed in patients with anti-TPO (negative 0.153 ± 0.216, positive 0.173 ± 0.274 ng/mL; *p* = 0.980) or anti-TG (negative 0.105 ± 0.071, positive 0.072 ± 0.033 ng/mL; *p* = 0.396) antibodies.

Concerning the potential relation of circulating GDF11 levels with cardiac function, no significant differences in GDF11 concentrations were found regarding ECG (normal 0.129 ± 0.185, anomalous 0.204 ± 0.339 ng/mL; *p* = 0.348), hypertension (no HTA 0.124 ± 0.177, HTA 0.137 ± 0.207 ng/mL; *p* = 0.567), or cardiopathy (negative 0.156 ± 0.262, positive 0.139 ± 0.152 ng/mL; *p* = 0.830). No significant correlation was observed between GDF11 levels and resting heart rate (*r* = −0.026, *p* = 0.791) or ejection fraction (*r* = −0.131, *p* = 0.413).

## 4. Discussion

The major finding of the present study is that circulating concentrations of GDF11 are closely associated with TSH concentrations and reduced in subjects with low TSH levels, but are apparently unrelated to REE. Other members of the TGF-β superfamily, such as GDF15 or GDF8, have been involved in energy homeostasis [21,22]. In the present study we observed a lack of correlation between the circulating concentrations of GDF11 and REE, which suggest that GDF11 is not involved in energy homeostasis. In agreement with this finding, we have previously observed that there are no differences in serum GDF11 concentrations in obese subjects as compared to lean ones [13], although GDF11 levels are correlated with BMI and waist circumference. It is possible that the association of GFD11 levels with BMI may be due to its potential involvement in the regulation of skeletal muscle mass. However, our recent findings are against this argument [13] and in the present work we have also found no relation of GDF11 levels with FFM or FFMI as indirect surrogates of skeletal muscle mass. Larger and interventional studies are needed to fully clarify whether or not GDF11 may play a role in energy expenditure regulation in humans.

We have found a strong positive correlation between the serum concentrations of GDF11 and the levels of TSH. Our work represents the first evidence of a relation between circulating levels of GDF11 and thyroid metabolism. Although correlation does not imply causality, two potential hypotheses arise from our finding. Firstly, that TSH may be positively regulated by GDF11. In this sense, several hormones and cytokines have been reported to affect TSH secretion. Cholecystokinin, interleukin (IL)-1β, tumor necrosis factor (TNF)-α, and neuropeptide Y (NPY) exert inhibitory effects, while GLP-1 and leptin, among others, stimulate TSH secretion [2,23]. Although the precise pathophysiological role of these factors remains to be elucidated, it has been suggested that they may play a role linking the nutrition and growth status with thyroid function [2,23]. Secondly, it seems plausible that GDF11 may be positively controlled by TSH or other thyroid hormones. No evidence has been reported regarding the effect of TSH or thyroxine on GDF11 levels or on the levels of any other GDF. TSH stimulates the thyroid gland to secrete thyroxine but, besides its effect on the thyroid gland, TSH also exerts actions in other tissues such as adipose tissue. In this sense, TSH has been involved in the regulation of lipolysis in white adipose tissue as well as in the control of fuel availability for BAT in the fasting- and cold-induced thermogenesis [3]. We did not observe different GDF11 levels in individuals taking L-thyroxine or a positive correlation of GDF11 levels with fT_4_, in agreement with previous work performed in mice [24]. Muscle dysfunction and atrophy are well known complications of thyrotoxicosis [25]. Therefore, if GDF11 has skeletal muscle regenerating capacity, one would expect high, rather than low, levels of GDF11 in patients with low levels of TSH, the opposite to what we observed. Alternatively, it can be speculated that the muscle dysfunction observed with thyrotoxicosis may be in relation with a deficient response in GDF11 secretion. Our data suggest that GDF11 is involved in thyroid metabolism although further in vitro and in vivo studies are needed to confirm this finding.

We observed a correlation between GDF11 levels and circulating leptin concentrations that was maintained after adjustment by age. Furthermore, in the multiple regression model, leptin together with TSH were significant predictors of GDF11 levels. Since GDF11 has no apparent relation with the control of fat mass nor is it expressed by this tissue, it can be speculated that GDF11 expression could be regulated by leptin. However, the significance of leptin as predictor of GDF11 levels was no longer observed when BMI, age, and CRP were also included in the multiple regression model.

Initial studies suggesting a rejuvenating action for GDF11 showed that after parabiosis experiments in mice, reversal of age-related cardiac hypertrophy was due to a blood-borne factor that was attributed to GDF11 [26,27]. However, other reports have either failed to confirm these findings [28] or showed important side effects of GDF11 in the heart [29]. In the present study we have observed that GDF11 levels are not related to the presence of an anomalous ECG, hypertension, or cardiopathy. Moreover, GDF11 concentrations were not associated with resting heart rate or ejection fraction. Although higher GDF11 levels have been associated with lower risk of cardiovascular events and death in the Heart and Soul Study [9] further mechanistic studies and analysis in larger cohorts are necessary to elucidate whether or not GDF11 levels play a role in cardiovascular function.

The association of circulating concentrations of GDF11 with CRP levels suggests that this factor may be involved in the regulation of inflammation or the acute-phase response. GDF11 expression could be induced in response to inflammation. In this sense, it has been reported that GDF11 opposes TNF-α-induced inflammatory actions protecting against the development of inflammatory arthritis in mice [30]. In addition, GDF11, by inhibiting the NLRP3 inflammasome activation evidenced in vivo and in vitro, ameliorates experimental colitis in mice [31]. Further studies aimed to assess the role of GDF11 in inflammatory conditions will clarify this finding.

One limitation of our study is that it was performed in Caucasian individuals and it would need to be confirmed whether our findings extend to other populations. Another limitation is that we did not measure TSH receptor-stimulating immunoglobulins that could be influencing GDF11 secretion. Further studies may elucidate the potential role of antibodies affecting TSH receptor in the regulation of GDF11 levels.

In conclusion, our results show that circulating concentrations of GDF11 are closely associated with TSH concentrations and reduced in subjects with low TSH levels. However, GDF11 is not related to the regulation of energy expenditure. Our data also suggest that GDF11 may be involved in the regulation of inflammation. Further studies modulating GDF11 levels and activity will undoubtedly help to elucidate the exact role of GDF11 in metabolism and its potential involvement in thyroid pathophysiology.

## Figures and Tables

**Table 1:** Demographic, biochemical, and metabolic characteristics of the individuals included in the overall study.

	All	Male	Female	*p*
*n*	287	121	166	
Age, year	50 ± 16	50 ± 17	50 ± 16	0.944
Weight, kg	101 ± 27	116 ± 26	90 ± 23	<0.001
BMI, kg/m^2^	36.3 ± 8.5	38.3 ± 8.1	34.8 ± 8.4	<0.001
Body fat, %	45.3 ± 8.8	41.8 ± 8.2	47.9 ± 8.4	<0.001
FFM, kg	54.0 ± 12.7	65.7 ± 9.2	45.4 ± 6.6	<0.001
FFMI, kg/m^2^	19.3 ± 3.1	21.7 ± 2.5	17.5 ± 2.1	<0.001
Waist circumference, cm	114 ± 20	123 ± 17	106 ± 18	<0.001
SBP, mm Hg	127 ± 18	132 ± 16	123 ± 18	<0.001
DBP, mm Hg	79 ± 11	83 ± 11	75 ± 9	<0.001
REE, kcal/d	1860 ± 421	2141 ± 355	1619 ± 309	<0.001
REE/FFM, kcal/d/kg	34.29 ± 3.77	33.92 ± 3.42	35.45 ± 3.68	<0.001
Glucose, mg/dL	104 ± 23	109 ± 24	100 ± 21	0.002
Insulin, μU/mL	15.8 ± 13.6	18.6 ± 11.3	13.8 ± 14.7	0.004
HOMA	4.3 ± 4.3	5.3 ± 4.4	3.5 ± 4.1	<0.001
Triglycerides, mg/dL	119 ± 69	137 ± 65	107 ± 70	<0.001
Total cholesterol, mg/dL	197 ± 40	192 ± 45	200 ± 35	0.150
LDL-cholesterol, mg/dL	120 ± 37	119 ± 42	120 ± 32	0.948
HDL-cholesterol, mg/dL	53.3 ± 16.4	45.7 ± 10.9	58.9 ± 17.6	<0.001
Uric acid, mg/dL	5.7 ± 1.7	6.9 ± 1.4	4.9 ± 1.3	<0.001
CRP, mg/L	8.7 ± 8.1	7.5 ± 8.54	9.6 ± 7.6	0.043
Creatinine, mg/dL	0.83 ± 0.22	0.97 ± 0.21	0.72 ± 0.16	<0.001
Leptin, ng/mL	37.3 ± 27.1	26.1 ± 16.3	45.1 ± 30.3	<0.001
TSH, μU/mL	3.6 ± 11.1	2.5 ± 2.8	4.4 ± 14.3	0.794
GDF11, ng/mL	0.121 ± 0.171	0.129 ± 0.199	0.114 ± 0.148	0.986

Data presented as mean ± SD. BMI: Body mass index; FFM: Fat-free mass; FFMI: Fat-free mass index; SBP: Systolic blood pressure; DBP: Diastolic blood pressure; REE: Resting energy expenditure; HOMA: Homeostatic model of assessment; CRP: C-reactive protein; TSH: Thyroid-stimulating hormone; GDF11: Growth differentiation factor 11; LDL: Low-density lipoprotein; HDL: High-density lipoprotein. Differences between groups were analyzed by two-tailed unpaired Student’s *t* tests. Triglycerides, CRP, leptin, TSH, and GDF11 concentrations were logarithmically transformed for statistical analysis due to their non-normal distribution.

**Table 2:** Univariate analysis of the correlation between GDF11 and other variables, unadjusted and after adjusting for age.

	Serum logGDF11			
	Unadjusted Correlation		Adjusted Correlation	
Variable	*r*	*p* Value	*r*	*p* Value
Sex	−0.01	0.986	−0.04	0.995
Age	**−0.16**	**0.006**	—	—
Weight	**0.14**	**0.018**	0.10	0.084
BMI	**0.15**	**0.009**	**0.14**	**0.021**
Body fat	0.11	0.076	0.11	0.058
FFM	0.08	0.206	0.03	0.594
FFMI	0.10	0.094	0.08	0.212
Waist circumference	**0.13**	**0.029**	**0.13**	**0.031**
SBP	0.05	0.402	0.10	0.116
DBP	0.07	0.279	0.07	0.221
REE	0.10	0.149	0.05	0.509
REE/FFM	0.02	0.725	−0.01	0.887
Glucose	0.01	0.969	0.04	0.544
Insulin	0.07	0.278	0.06	0.304
HOMA	0.06	0.308	0.06	0.295
Triglycerides	0.03	0.629	0.04	0.487
Total cholesterol	0.05	0.407	0.06	0.298
LDL-cholesterol	0.07	0.265	0.07	0.239
HDL-cholesterol	−0.05	0.367	−0.04	0.505
Uric acid	0.04	0.498	0.03	0.571
logCRP	**0.19**	**0.025**	**0.18**	**0.038**
Creatinine	0.06	0.310	0.09	0.146
Leptin	**0.14**	**0.027**	**0.14**	**0.026**
logTSH	**0.40**	**<0.001**	**0.38**	**<0.001**

Values are Pearson’s correlation coefficients and associated *p* values. CRP and TSH concentrations were logarithmically transformed for statistical analysis. GDF11: Growth differentiation factor 11; BMI: Body mass index; FFM: Fat-free mass; FFMI: Fat-free mass index; SBP: Systolic blood pressure; DBP: Diastolic blood pressure; REE: Resting energy expenditure; HOMA: Homeostatic model assessment; CRP: C-reactive protein; TSH: Thyroid-stimulating hormone; LDL: Low-density lipoprotein; HDL: High-density lipoprotein. For correlation with gender, male = 1 and female = 2 was used. Bold values denote statistical significance at *p <* 0.05.

**Table 3:** Multiple regression analysis with logGDF11 as dependent variable.

Variable		*r* ^2^	*β*	*p* Value
**Model 1**		0.15		<0.001
	logTSH		0.397	<0.001
**Model 2**		0.23		<0.001
	logTSH		0.462	<0.001
	Leptin		0.119	0.037
**Model 3**		0.37		<0.001
	logTSH		0.558	<0.001
	Leptin		0.098	0.212
	BMI		0.079	0.336
	Age		−0.062	0.388
	logCRP		0.006	0.940

Values are corrected *r*^2^ (*r*^2^), standardized coefficients (*β*) and associated *p* values. GDF11: Growth differentiation factor 11; TSH: Thyroid-stimulating hormone; BMI: Body mass index; CRP: C-reactive protein.

**Table 4:** Demographic, biochemical, and metabolic characteristics of the individuals classified according to TSH circulating concentrations.

	High-TSH	Normal-TSH	Low-TSH	*p*
*n*	32	76	20	
Sex, M/F	11/21	31/45	6/14	0.617
Age, year	43 ± 13	45 ± 14	50 ± 8	0.142
Weight, kg	96 ± 26	97 ± 31	90 ± 21	0.612
BMI, kg/m^2^	34.7 ± 7.5	34.7 ± 9.7	32.0 ± 6.7	0.442
Body fat, %	43.9 ± 9.2	43.7 ± 8.8	43.1 ± 7.2	0.936
FFM, kg	52.9 ± 12.2	53.4 ± 13.9	51.2 ± 10.4	0.797
FFMI, kg/m^2^	19.0 ± 2.6	19.0 ± 3.4	18.1 ± 3.0	0.534
Waist circumference, cm	109 ± 19	109 ± 22	105 ± 15	0.689
SBP, mm Hg	120 ± 18	123 ± 16	118 ± 13	0.520
DBP, mm Hg	76 ± 9	76 ± 11	74 ± 8	0.712
REE, kcal/d	1713 ± 380	1799 ± 452	2007 ± 566	0.269
REE/FFM, kcal/d/kg	32.97 ± 3.76	33.71 ± 3.69	35.97 ± 3.68	0.136
Glucose, mg/dL	102 ± 30	102 ± 23	114 ± 31	0.201
Insulin, μU/mL	13.9 ± 12.0	15.4 ± 14.5	16.0 ± 10.0	0.846
HOMA	3.9 ± 4.6	4.2 ± 5.1	4.5 ± 2.8	0.907
Triglycerides, mg/dL	121 ± 70	110 ± 66	112 ± 50	0.644
Total cholesterol, mg/dL	214 ± 48	201 ± 39	178 ± 42 * †	0.018
LDL-cholesterol, mg/dL	136 ± 42	124 ± 38	109 ± 39	0.069
HDL-cholesterol, mg/dL	54.5 ± 16.4	54.8 ± 18.8	47.4 ± 11.7	0.263
Uric acid, mg/dL	5.7 ± 2.1	5.4 ± 1.7	4.7 ± 1.3	0.175
CRP, mg/L	10.9 ± 12.5	7.4 ± 7.5	5.6 ± 5.8	0.477
Creatinine, mg/dL	0.84 ± 0.23	0.79 ± 0.20	0.74 ± 0.21	0.265
Leptin, ng/mL	34.1 ± 21.7	35.1 ± 28.9	31.5 ± 26.9	0.531
TSH, µU/mL	17.3 ± 30.0	2.1 ± 1.0 *	0.2 ± 0.1*	<0.001
Thyroxine (fT_4_), pmol/L	13.0 ± 3.8	15.8 ± 2.6 *	18.9 ± 4.1 * †	<0.001
Triiodothyronine (fT_3_), pmol/L	2.56 ± 1.32	2.14 ± 1.14	2.05 ± 1.02	0.374
Heart rate, bpm	73 ± 14	75 ± 14	68 ± 13	0.873

Data presented as mean ± SD. BMI: Body mass index; FFM: Fat-free mass; FFMI: Fat-free mass index; SBP: Systolic blood pressure; DBP: Diastolic blood pressure; REE: Resting energy expenditure; HOMA: Homeostatic model assessment; CRP: C-reactive protein; TSH: Thyroid-stimulating hormone; LDL: Low-density lipoprotein; HDL: High-density lipoprotein; bpm: Beats per minute. Differences between groups were analyzed by ANOVA followed by LSD tests. * *p <* 0.05 vs. High-TSH. **†** *p <* 0.05 vs. Normal-TSH. Differences in gender distribution were analyzed by χ^2^ analysis. Triglycerides, CRP, leptin, TSH, and GDF11 concentrations were logarithmically transformed for statistical analysis due to their non-normal distribution.

## Abbreviations

BMI	body mass index
BMP	bone morphogenetic protein
bpm	beats per minute
CRP	C-reactive protein
DBP	diastolic blood pressure
ECLIA	electro-chemiluminescence immunoassay
ELISA	enzyme-linked immunosorbent assay
FFM	fat-free mass
FFMI	fat-free mass index
fT_3_	free triiodothyronine
fT_4_	free thyroxine
GDF8/11/15	growth differentiation factor8/11/15
GLP-1	glucagon-like peptide-1
HOMA	homeostasis model assessment
HTA	hypertension
IL-1β	interleukin-1β
NPY	neuropeptide Y
REE	resting energy expenditure
RIA	radioimmunoassay
SBP	systolic blood pressure
TGF-β	transforming growth factor-β
TSH	thyroid-stimulating hormone
TNF-α	tumor necrosis factor-α
